# Efficacy and safety of entacapone in levodopa/carbidopa versus levodopa/benserazide treated Parkinson’s disease patients with wearing-off

**DOI:** 10.1007/s00702-015-1449-6

**Published:** 2015-09-07

**Authors:** Mikko Kuoppamäki, Mika Leinonen, Werner Poewe

**Affiliations:** Orion Pharma/R&D, P.O. Box 425, 20101 Turku, Finland; 4Pharma AB, Stockholm, Sweden; Universitätsklinik für Neurologie, Leopold-Franzens-Universität, Innsbruck, Austria

**Keywords:** Parkinson’s disease, Levodopa, Carbidopa, Benserazide, Entacapone

## Abstract

Entacapone is frequently used together with levodopa/carbidopa (LC) and levodopa/benserazide (LB) in the treatment of Parkinson’s disease (PD) patients with wearing-off symptoms. It is generally assumed that the effects of entacapone are independent of the type of decarboxylase inhibitor used, but there is very little published data available on the efficacy of entacapone administered with LB versus LC. We have performed a pooled analysis of three randomized, double-blind, 6-month, phase III studies to compare the treatment effects of entacapone (compared to placebo) in PD patients receiving LC or LB. A total of 551 PD patients experiencing wearing-off were included in the analysis. 300 patients were on LB and 251 on LC at baseline. At 6 months, entacapone (compared to placebo) improved mean daily OFF-time in patients on LB and LC by 0.76 (*p* = 0.016) and 0.95 (*p* = 0.011) hours, respectively. The corresponding improvements in ON-time were 0.97 (*p* = 0.002) and 0.83 h (*p* = 0.022), respectively. The treatment effects of entacapone both in LB and LC users were statistically significant (*p* < 0.05) also in UPDRS II and III scores, except in UPDRS II scores in patients receiving LC (*p* = 0.20). None of the treatment effects of entacapone were statistically significantly different between patients receiving LB or LC. Reported adverse events were comparable between LB and LC users. We conclude that entacapone provided comparable benefits in PD patients with wearing-off symptoms using either LB or LC.

## Introduction

After more than 40 years of routine clinical use levodopa has remained the gold standard of symptomatic efficacy in the treatment of Parkinson’s disease (PD). Levodopa is always combined with a dopa-decarboxylate inhibitor (DDCI) and current preparations either use carbidopa or benserazide to block the main levodopa metabolizing enzyme. Earlier randomized, controlled studies in PD patients have reported that the clinical effects and levodopa pharmacokinetics (PK) are comparable between carbidopa and benserazide (Greenacre et al. 1976; Pakkenberg et al. 1976; Rinne and Mölsä 1979).

Adjunct therapy with a catechol-*O*-methyltransferase (COMT) inhibitor is a first-line strategy when treating PD patients who have developed wearing-off with levodopa/carbidopa (LC) or levodopa/benserazide (LB). Entacapone and tolcapone are the two COMT-inhibitors currently available. Both increase the bioavailability of levodopa combined with carbidopa (Keränen et al. 1993; Sedêk et al. 1997) and benserazide (Kaakkola et al. 1994; Dingemanse et al. 1995) and thereby prolonging clinical response (Nutt et al. 1994; Kaakkola et al. 1994, 1995; Baas et al. 1997).

Randomized, double-blind, placebo-controlled phase III trials have confirmed the efficacy of entacapone in reducing OFF-time in PD patients with wearing-off compared to placebo (PSG 1997; Rinne et al. 1998; Poewe et al. 2002; Brooks and Sagar 2003). In three of these studies (Rinne et al. 1998; Poewe et al. 2002; Brooks and Sagar 2003), patients were receiving either LC or LB while in the fourth study (PSG 1997) all patients received LC. These data were analysed for the entire study populations, not separately for patients on LC or LB, thus there are very little published data on the efficacy and safety of entacapone specifically in patients using LB. We were able to identify only one small study conducted in nine wearing-off patients (Kaakkola et al. 1994), which reported increased plasma AUC of levodopa and improved motor disability after starting entacapone as add-on to each dose of LB.

Here we have pooled data from three phase III entacapone trials, which recruited patients both on LC or LB. The aim of the analysis was to evaluate the efficacy and safety of entacapone (compared with placebo) separately for patients receiving LC and LB and to compare the treatment effect of entacapone between patients on the two DDC-inhibitors.

## Materials and methods

This was a retrospective, pooled analysis of three randomized, double-blind, placebo-controlled, 6-month, phase III studies in PD patients with wearing-off (Rinne et al. 1998; Poewe et al. 2002; Brooks and Sagar 2003 including patients both on LC or LB. Patients’ antiparkinsonian medications (especially levodopa) were individually optimized before randomization. Other antiparkinsonian medications were allowed, but doses were not to be changed during the studies. The phase III study conducted by the Parkinson Study Group (PSG 1997) was left out from the dataset, because it did not include any patients receiving LB.

A pooled analysis is similar to a traditional meta-analysis, however, outcome measures and other data are combined from multiple studies and are analysed as a single dataset. This pooled analysis was possible due to access to the raw data from studies with similar study design and efficacy variables (ON/OFF-time by home diary and UPDRS scores). Two of the original studies also included non-fluctuating patients (Poewe et al. 2002; Brooks and Sagar 2003) and these patients were excluded from the present dataset. There was a small number of patients using both LC and LB (mixed users) and they were also excluded from the analysis. In all studies, patients on LC or LB were randomized to receive either entacapone or placebo.

Efficacy variables were daily ON- and OFF-times recorded by patient diary (collected as 18 or 24 h diaries and standardized to18 h) and Unified Parkinson’s Disease Ratings Scale (UPDRS) part II (ADL) and III (motor) scores. The original studies were conducted in 1993–1998. During that time, patient diaries included only variables of ‘ON’, ‘OFF’ or ‘IN BED’. UPDRS III scores were assessed during ON-state and UPDRS II scores reflected patient’s daily functioning over a preceding week without specifying between ON- and OFF- states. ON- and OFF-times, UPDRS scores and mean daily levodopa doses were analysed using the intention-to-treat datasets of each original study with observed cases (ITT-OC). Analysis of covariance (ANCOVA) models with fixed effects for treatment (entacapone or placebo), DDCI group (LB or LC) and their interaction were used to estimate the difference between entacapone and placebo within the LB and LC treated patients and the difference in the treatment effect (entacapone vs placebo) between the LB and LC treated patients. Baseline value was used as a covariate. All efficacy results are reported as changes from baseline at 6 months (24 weeks) estimated with the ANCOVA model. No correction for multiple comparisons was done. All the statistical analyses were performed using the SAS^®^ software version 9 (SAS Institute Inc., Cary, NC, USA).

The safety was evaluated by adverse events (AEs). All AEs were coded with World Health Organization Adverse Reactions Terminology (WHO-ART) and the proportions of patients in each group reporting treatment-emergent AEs up to 6 months were summarized by the preferred term. All AEs reported in at least one group by at least 3 % of the patients are reported. The incidences of AEs were analysed using a logistic regression model including terms for treatment group (entacapone or placebo), DDCI (LB or LC) and their interaction.

Each original study was performed according to good clinical practice, reviewed by the local ethics committee and approved by the relevant national competent authorities. All subjects gave their informed consent.

## Results

### Baseline demographics

A total of 551 PD patients experiencing wearing-off were included in the ITT analysis, of which 300 patients were on LB and 251on LC at baseline. A total of 336 and 215 patients were randomized to entacapone and placebo, respectively. In the LB group, 170 and 130 patients were randomized to entacapone and placebo, respectively. The respective numbers of randomized patients in the LC group were 166 and 85. The safety dataset was identical to the dataset used for the ITT analysis.

At baseline in the total study population, the mean (SD) age was 62.8 (9.3) years, duration of PD 9.6 (5.0) years, duration of levodopa treatment 8.0 (4.6) years, daily levodopa dose 646 (328) mg and daily OFF-time 5.9 (2.4) h. There were no significant differences in baseline characteristics between patients receiving LB or LC (Table 1).

**Table 1:** Demographics and baseline data for patients randomized to entacapone or placebo and using levodopa/benserazide (LB) or levodopa/carbidopa (LC)

	LB		LC		Total (*N* = 551)
	LB and entacapone (*N* = 170)	LB and placebo (*N* = 130)	LC and entacapone (*N* = 166)	LC and placebo (*N* = 85)	
Age (years)	61.8 (9.0)	62.3 (9.2)	63.9 (9.5)	63.3 (9.5)	62.8 (9.3)
Gender (male/female, %)	55/45	54/46	63/37	62/38	58/42
Duration of Parkinson’s Disease (years)	9.2 (5.1)	10.4 (5.1)	9.0 (4.9)	10.2 (5.1)	9.6 (5.0)
Duration of L-dopa treatment (years)	7.6 (4.4)	8.5 (4.4)	7.6 (4.6)	8.7 (4.9)	8.0 (4.6)
Hoehn and Yahr staging (≤2/≥2.5, %)	45/55	52/48	37/63	39/61	43/57
Levodopa dose (mg)	626 (320)	630 (289)	645 (327)	717 (391)	646 (328)
Dopamine agonist users (%)	56	55	60	60	57
MAO-B inhibitor users (%)	48	49	45	41	46
Amantadine users (%)	13	14	21	13	15
Anticholinergic users (%)	17	15	13	17	15
Other antiparkinsonian medication users (%)	1	1	1	0	1
OFF time (h)	6.1 (2.4)	5.8 (2.5)	5.6 (2.3)	5.8 (2.5)	5.9 (2.4)
UPDRS part III at ON state	25.1 (13.1)	24.3 (12.4)	23.8 (12.0)	24.3 (12.1)	24.4 (12.5)

### Efficacy

**Fig. 1:**
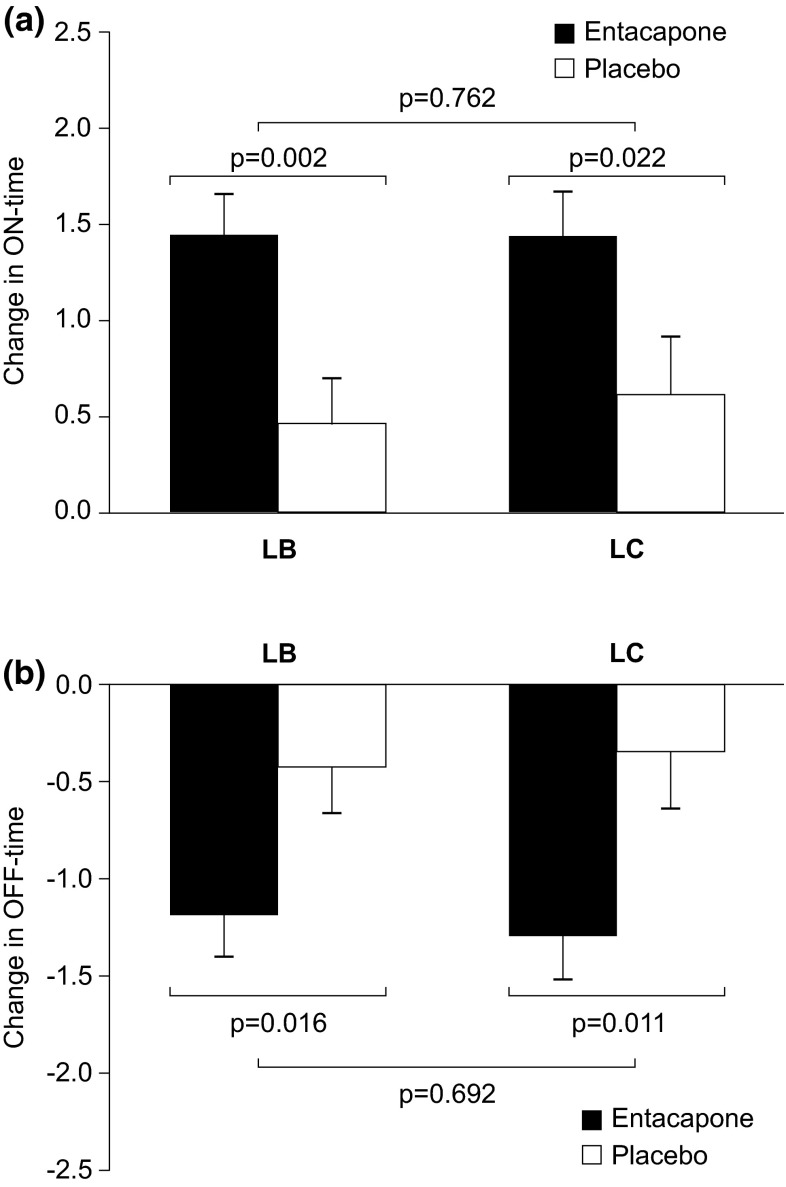
Changes in daily ON- and OFF-times in patients randomized to entacapone or placebo and using levodopa/benserazide (LB) or levodopa/carbidopa (LC). **a** ON-time and **b** OFF-time. *Bars* represent estimated mean changes with standard error of mean (SEM); ITT-OC (intent to treat observed cases). *Black bars* entacapone; *white bars* placebo

At 6 months, a significant decrease in mean OFF-time in LB (−0.76 h; 95 % confidence interval (CI) from −1.37 to −0.15 h; *p* = 0.016) and LC (−0.95 h; 95 % CI from −1.67 to −0.23 h; *p* = 0.011) groups was seen in patients randomized to entacapone compared with those receiving placebo. The respective increases in ON-time were 0.98 h (0.36–1.59 h; *p* = 0.002) and 0.82 h (0.09–1.55 h; *p* = 0.022) for LB and LC, respectively. Treatment effects of entacapone did not differ significantly between patients on LB or LC regarding OFF- (*p* = 0.692) and ON-times (*p* = 0.762) (Fig. 1).

**Fig. 2:**
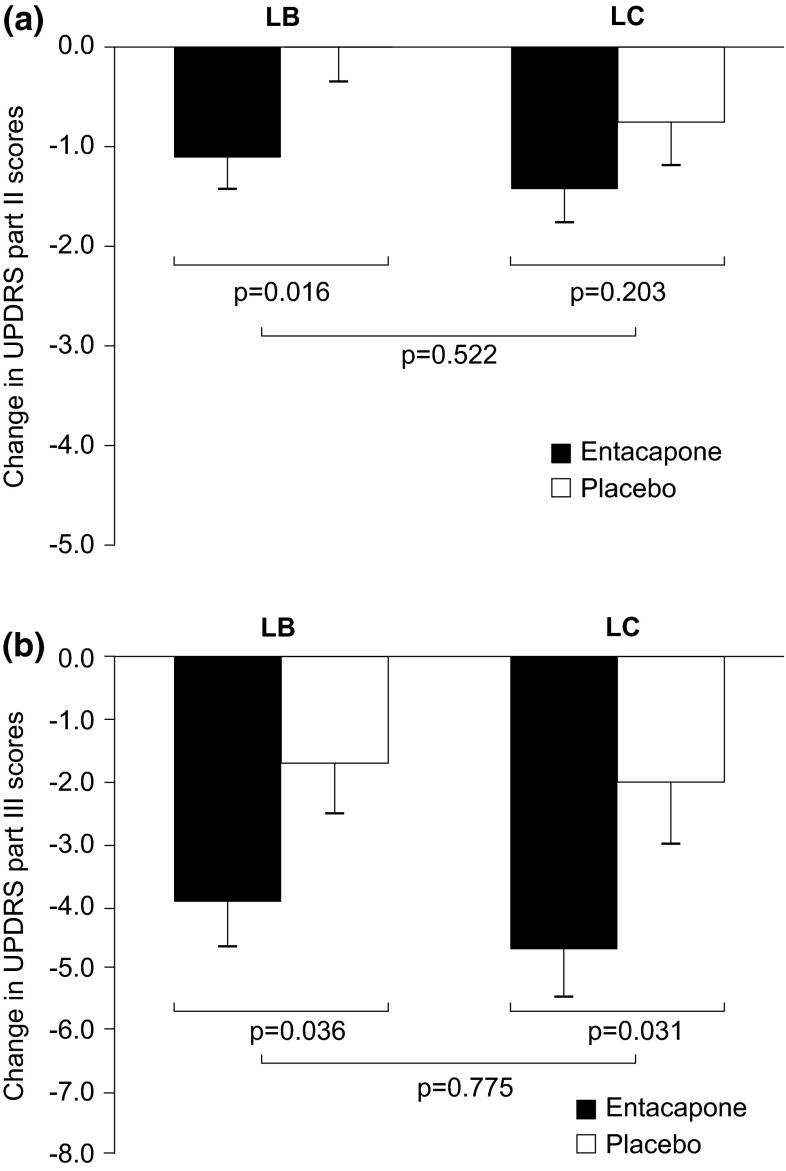
Changes in UPDRS II and III scores in patients randomized to entacapone or placebo and using levodopa/benserazide (LB) or levodopa/carbidopa (LC). **a** UPDRS II and **b** UPDRS III. *Bars* represent estimated mean changes with standard error of mean (SEM); ITT-OC (intent to treat observed cases). *Black bars* entacapone; *white bars* placebo. *UPDRS* Unified Parkinson’s Disease Rating Scale

The improvements in mean (95 % CI) UPDRS part II scores in patients receiving entacapone were −1.1 (−2.0 to −0.2); *p* = 0.016) and −0.7 (−1.7 to 0.4); *p* = 0.203) compared with placebo in patients on LB and LC, respectively. In turn, entacapone improved mean (95 % CI) UPDRS part III scores by −2.2 points (−4.2 to −0.2); *p* = 0.036) and −2.6 points (−5.0 to −0.3); *p* = 0.031) compared with placebo in LB and LC users, respectively. The treatment effects of entacapone in UPDRS part II (*p* = 0.522) and part III (*p* = 0.775) scores were not different between LB and LC users (Fig. 2).

Mean (95 % CI) daily levodopa dose decreased significantly during the 6-month follow-up in LB and LC patients randomized to entacapone by −82 mg (−120 to −45); *p* < 0.001) and −52 mg (−95 to −9); *p* = 0.020), respectively. The treatment effects were not significantly different (*p* = 0.294) between patients on LB and LC.

### Tolerability and safety

Entacapone was generally well tolerated in patients on LB or LC. The most commonly reported AEs (preferred term) for entacapone were dyskinesia, diarrhea, hyperkinesia, nausea, constipation and hypokinesia. Among those receiving entacapone, 4 patients (2.4 %) on LB and and 3 patients (1.8 %) on LC discontinued the study due to diarrhea. In the placebo group, the respective numbers were 1 (0.8 %) and 0 patients. All the AEs reported at least in 3 % of patients in the entire study population are presented in Table 2. No statistically significant interactions between entacapone treatment and DDCI were seen for the commonly reported AEs.

**Table 2:** Adverse events in patients receiving entacapone or placebo with levodopa/benserazide (LB) or levodopa/carbidopa (LC) reported in at least one group by ≥3 % of the patients

Preferred term	LB and entacapone (*N* = 170) %	LB and placebo (*N* = 130) %	LC and entacapone (*N* = 166) %	LC and placebo (*N* = 85) %	*p* value (interaction between treatment and DDCI)
Dyskinesia	16	8	17	8	0.997
Diarrhoea	13	5	11	7	0.341
Hyperkinesia	11	5	15	9	0.516
Nausea	11	6	16	6	0.472
Constipation	11	3	4	4	0.158
Hypokinesia	10	5	11	11	0.253
Parkinsonism aggravated	10	8	9	17	0.091
Abdominal pain	8	5	6	6	0.638
Dizziness	7	5	6	1	0.295
Urine abnormal	7	0	11	0	–
Insomnia	4	5	4	6	0.912
Hallucination	3	5	4	5	0.557
Dystonia	1	4	3	7	0.762

## Discussion

This is the largest pooled analysis of the efficacy and safety of entacapone specifically in patients on LB including a total of 300 patients of which 170 and 130 were randomized to entacapone and placebo, respectively. Entacapone significantly improved daily OFF- and ON-times as well as UPDRS II and III scores irrespective of the DDCI (carbidopa or benserazide) combined with levodopa. These results are well in line with separate reports and the overall pooled analysis of entacapone phase III studies (PSG 1997; Rinne et al. 1998; Poewe et al. 2002; Brooks and Sagar 2003; Kuoppamäki et al. 2014). An important finding was also that the treatment effects of entacapone (vs placebo) did not differ between patients on LC or LB. In other words, patients on LB received similar benefits of entacapone for the treatment of their wearing-off symptoms compared to those on LC. As the present analysis was based on data from a pooled dataset, the analysis was not prospectively powered for hypothesis testing and can therefore be considered as explorative. For the comparison of OFF-time difference between the two DDCIs, the analysis had 92 % power to show a difference of 1.5 h in daily OFF-time as statistically significant with a two-sided significance level of 0.05 and assuming the observed standard deviation of 2.5 h based on a test for the interaction term between randomized treatment group and DDCI. This suggests that despite lack of a prospective sample size calculation, the analysis is adequately powered to detect clinically meaningful treatment effects. Tolerability and safety of entacapone was also comparable between patients on LC or LB.

Entacapone has become available as a separate product and as a triple combination of levodopa/carbidopa/entacapone (LCE), the latter type of combination not being available with benserazide. Nevertheless, an open-label, 6-week study has reported that patients on LB experienced clinically relevant improvements in their condition when switching directly from LB to LCE. The study also found that the efficacy and safety of switching from LB to LCE was comparable with switching from LC to LCE (Eggert et al. 2010). This finding is also in agreement with our present analysis.

In conclusion, using a pooled analysis of three phase III entacapone studies, we are reporting that adding entacapone provided comparable benefits to PD patients on either LC or LB when treating their wearing-off symptoms. To our knowledge, this is the largest dataset reporting the efficacy and safety of entacapone in PD patients receiving LB.
